# Effects of Electroacupuncture on Benign Prostate Hyperplasia Patients with Lower Urinary Tract Symptoms: A Single-Blinded, Randomized Controlled Trial

**DOI:** 10.1155/2011/303198

**Published:** 2011-04-13

**Authors:** Jung-Sheng Yu, Kun-Hung Shen, Wen-Chi Chen, Jiann-Shyan Her, Ching-Liang Hsieh

**Affiliations:** ^1^Department of Traditional Chinese Medicine, Chi Mei Medical Center, Tainan 71004, Taiwan; ^2^Graduate Institute of Integrated Medicine, China Medical University, Taichung 40402, Taiwan; ^3^Division of Urology, Department of Surgery, Chi Mei Medical Center, Tainan 71004, Taiwan; ^4^Graduate Institute of Acupuncture Science, China Medical University, 91 Hsueh-Shih Road, Taichung 40402, Taiwan; ^5^Acupuncture Research Center, China Medical University, Taichung 40402, Taiwan; ^6^Department of Chinese Medicine, China Medical University Hospital, Taichung 40402, Taiwan

## Abstract

We tested the effect of electroacupuncture (EA) on lower urinary tract symptoms (LUTS) in benign prostatic hyperplasia (BPH) patients. A total of 42 BPH patients with LUTS were randomly assigned to either the EA group (EG), received 2 Hz EA for 20 min twice/week for a total of twelve treatments, or a sham EA group (CG), received sham EA. The increase of voiding volume, average flow rate, and maximal flow rate in the EG were 32.2 ± 104.4 mL, 1.2 ± 1.6 mL/sec, and 2.3 ± 3.7 mL/sec, respectively, from baseline value (before EA) using the measurement of an uroflowmetry. These increases were greater than −37.9 ± 120.4, −0.22 ± 2.7, and −0.3 ± 4.3, respectively, in the CG (*P* = .038, .026, and .030, resp.). The changes of prostate special antigen and international prostatic symptom score were not significantly different between two groups (*P* = .573, .175, resp.), suggesting the clinical improvement of 2 Hz EA was quite limited to the LUTS of patients with BPH.

## 1. Introduction

Benign prostate hyperplasia (BPH) is a common condition in men over 50 years of age. BPH and prostate size increase continuously with age [1]. Sommer et al. (1990) found that the prevalence and the severity of voiding symptoms including obstructive and irritative symptoms increased in men between 50 and 60 years of age, suggesting a relationship to BPH [2]. The prevalence BPH is 20.2% in men 40–64 years of age and 42.8% in men 65–79 years of age in the community in central Scotland [3]. The symptoms of BPH includes obstructive symptoms of weak urinary stream force, hesitancy, intermittent, terminal dribbling and incomplete bladder emptying, and irritative symptoms of frequency, nocturia, and urgency. These symptoms may affect quality of life [3–5]. The symptoms of BPH increase with age in Japanese men, as they do in men in the United States [6]. It has been observed that prostate size does not correspond to the severity of obstruction and other symptoms [7]. The international prostate symptom score (IPSS) scoring system has been developed by the American Urological Association [8]. This scores system, from 0 to 35, covers mild, moderate, and severe lower urinary symptoms, and includes seven questions relating to voiding and filling symptoms in the scoring system. The IPSS is a sensible and reliable system clinically [8] and has been used widely in the clinic for the evaluation of the severity of lower urinary tract symptoms (LUTS) in patients with BPH [5, 9, 10]. Uroflowmetry is one of the simplest urodynamic methods and is noninvasive and, thus, plays a critical role in the assessment of obstructive LUTS in patients with BPH [11]. Uroflowmetry together with symptomatology constitute a reliable method for preoperative evaluation in patients with BPH [12]. That the maximum flow rate (Qmax), average flow rate (Qave), and volume of voided urine are lower in patients with BPH and that the total flow time and time to start voiding are longer than those of healthy men have been reported in a study using uroflowmetry [13]. Therefore, uroflowmetry may be used as an index to evaluate the severity of symptoms in patients with BPH. Prostate specific antigen (PSA) is an important tumor marker of prostate cancer and has been widely used in the early diagnosis and management of patients with BPH or with prostate cancer [14, 15]. 

Although nonselective alpha blockers such as Doxaosin or Prazosin and selective alpha blocker such as Tamsulosin can mitigate LUTS in patients with BPH, these drugs may cause side effect of postural hypotension fatigue and dizziness, [5, 16]. Finasteride as an alpha reductase inhibitor may decrease the volume of BPH and also may decrease the rate of hematuria and prostate cancer, but it may result in sexual dysfunction [5, 17]. Acupuncture has been used to treat LUTS for thousands of years. Acupuncture treatment achieves greater reduction in frequency and urgency and results in greater increase in functional bladder capacity and greater improvement in the score of an incontinence impact questionnaire than does placebo treatment in women with overactive bladder [18]. Acupuncture also may reduce the National Institutes of Health chronic prostatitis symptom index (NIH-CPSI) in total score and pain score, as well as urinary and quality-of-life scores in patients with chronic prostatitis/chronic pelvic pain syndrome [19]. Johnstone et al. (2003) reported that acupuncture cannot relieve LUTS and PSA in BPH patients [20]. In contrast, others have found that electroacupuncture (EA) at Zhongji (CV3) may significantly improve Qmax, frequency of nocturia, and urine steam [21], and EA at Zhonji and Huiyin (CV1) may improve Qmax and postvoided residual urine [22]. Therefore, the effect of EA on the LUTS in BPH patients needed further study. We designed a single-blinded, randomized pilot study, and used uroflowmetry, IPSS, and PSA as indices.

## 2. Material and Methods

### 2.1. Subjects

A total of 91 men with BPH, who suffered from LUTS including incomplete empting, frequency, intermittency, urgency, straining, and nocturia were enrolled from December 2008 to December 2009 at Chi Mei Medical Center (Tainan, Taiwan). Forty-seven patients were excluded, and two patients refused to participate in the trial. Forty-two men with BPH participated in the present study. The study protocol was approved by the institutional review board of Chi Mei Medical Center (IRB no. 09712-001). The procedures of the trial were in accord with *the ethical principles dictated in the Declaration of Helsinki*, and the informed consent form was signed by each participant after detailed explanation of the trial purpose and procedure. 

BPH was confirmed by a urology specialist using the transrectal sonography survey, and PSA also was measured for exclusion of prostate cancer. The inclusion criteria included (1) age > 40, (2) BPH in the absence of any previous anti-BPH treatment such as alpha blocker medications, or surgical operation, (3) IPSS > 8, and (4) Qmax < 15 mL/sec. The exclusion criteria were (1) finding that the lower urinary symptoms were due to interstitial cystitis, prostate cancer, urinary stone, or urinary tract infection, (2) any medical condition such as congestive heart failure, hypertensive patients treated with beta blockers, arrhythmia with or without cardiac pacemaker, chronic pulmonary obstructive disease, and hepatic failure, and (3) inability of the potential participant to comply with the schedule of the trial.

### 2.2. Randomization and Blinding

**Figure 1:**
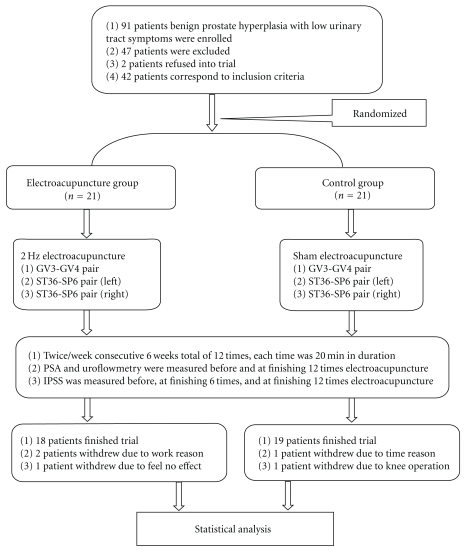
Flowchart.

Forty-two patients were randomized by lottery to the EA group (EG), which received EA, or to the control group (CG), which received sham EA. Each group had 21 subjects (Figure 1).

### 2.3. Study Design and Sample Size

The present study was a pilot study of a single-blind randomized controlled trial. The sample size was calculated according to Koseoglu et al. (2006) [23] and Loh et al. (2009) [9]. We predicted a dropout rate of 15%; therefore, twenty-one subjects would be sufficient.

### 2.4. EA

EA was performed by a Chinese medical doctor with more than 4 years of acupuncture experience. In the EG, the subjects were treated with stainless steel acupuncture disposable needles (2 cun in length, gauge #30, Yu Kuang, Taiwan) inserted into the Zhongji (CV3) and Guanyuan (CV4) and bilateral Zusanli (ST36) and Sanyinjiao (SP6) acupoints. The acupuncture needles were twisted manually 3–5 times to obtain qi (in which the acupuncturist has the sensation of fish biting on bait; the subject experiences a hot and tightness feeling). The Zhongji, Guanyuan, Zusanli, and Sanyijiao needles were connected to electroacupuncture apparatus (HC-0501, Hung-Tai Co., Taiwan). 

 Three pairs of EA were designed as Zhongji and Guanyuan, ipsilateral Zusanli and Sanyinjiao. These acupoints were chosen according to the selection of local points and according to meridian theory of Traditional Chinese Medicine. Zhongji and Guanyuan acupoints are located in the midline of the low abdomen, and 4 cun and 3 cun, respectively, inferior to the umbilicus, and belong to the conception vessel near the urinary bladder. Both acupoints may treat urinary disease including frequency, urgency, and dribbling [24]. The Zusanli acupoint is located 3 cun below the knee, belongs to the stomach meridian, and the acupoint may treat difficult urination [25]. The Sanyijiao acupoint is located 3 cun above the medial malleolus and belongs to the spleen meridian, and the acupoint may treat uroschesis and stranguria [26].

The frequency of EA was 2 Hz. The intensity of electrical stimulation was adjusted to obtain visible twitching of muscle about 2–2.5 mA. The subject did not feel pain or discomfort. The duration of electrical stimulation was 20 min and was performed two times per week for 6 consecutive weeks for a total of twelve sessions. In the CG, the methods were identical to those in the EG, except that the acupuncture needles were inserted into the subcutaneous tissue to a depth of 2 mm, the location was 1 cm lateral to the above-mentioned acupoints, respectively, without manual twisting or any electrical stimulation. The checklist of consolidated standards of reporting trials (CONSORT) was completed [27] and complete details of the intervention are presented in Table 1 in conformance to the standards for reporting interventions in controlled trial of acupuncture [28].

### 2.5. Outcome Measures

The primary outcome measures were the differences of Qmax, average flow rate (Qave), total flow time, and void volume from baseline (before EA) to after completion of twelve rounds of EA (F2). Those indices were measured using uroflowmetry (UROCOMPACT 6000, LABORIE, ENCORE).

The secondary outcome measures were the change of serum PSA concentration measured baseline and at F2, and the differences of IPSS includs incomplete empting, intermittency, weak stream, straining, frequency, urgency, and nocturia from baseline to after completion of six rounds of EA (F1), and from baseline to F2.

### 2.6. Statistical Analysis

The data are presented as mean ± standard deviation (SD) and are analyzed by SPSS for Windows, version 17.0 (SPSS Inc., Illinois, USA). Mann-Whitney U test was used to assess the differences between two groups. The significance level was set at *α* = 0.05. *P* < .05 was considered to indicate a statistically significant difference.

## 3. Results

### 3.1. Basic Characteristics

Forty-two BPH patients with LUTS participated in this trial. Three patients withdrew in the EG, two patients withdrew because of work, and one patient because he felt no effect. Two patients withdrew in the CG, one patient withdrew because of the time commitment and one patient because of a knee operation. Therefore, a total of 37 subjects completed the trial (Figure 1). No significant differences were found between the EG and CG in basic characteristics including age, prostate volume, IPSS, voiding volume, total flow time, Qave, Qmax, and serum PSA levels (Table 2).

 Three patients in the EG developed subcutaneous ecchymosis in the lower abdominal region. These ecchymoses were all less than 3 cm in diameter and disappeared spontaneously without any treatment. No one withdrew from the trial for this reason.

### 3.2. Effect of EA on Voiding Symptoms in BPH Patients

The increases in void volume, Qave, and Qmax from baseline to F2 were greater in the EG than in the CG (*P* = .038,   .026,  .030, resp.; Table 2), while the increase of total flow time was not significantly different between EG and CG (*P* = .607; Table 3).

### 3.3. Effect of EA on LUTS and PSA in BPH Patients

The changes of IPSS from baseline to F1, and from baseline to F2 were not significantly different between EG and CG (*P* = .314,   .175, resp., Table 4).

The changes of serum PSA levels from baseline to F2 was not significantly different between EG and CG (*P* = .573; Table 4).

The changes of subscore of IPSS including incomplete empting, frequency, intermittency, urgency, weak stream, straining, and nocturia from baseline to F1 and from baseline to F2 were not significantly different between EG and CG (Table 5).

## 4. Discussion

The results in the present study indicated that increases in voiding volume, Qave, and Qmax were greater in the EG than in the CG in BPH patients with LUTS, whereas total flow time, and IPSS and subscore of IPSS in the EG was similar to that in the CG. These results suggested that the clinical improvement of 2 Hz EA was quite limited to the LUTS of patients with BPH. The 2 Hz EA may enhance the release of enkephalin, *β*-endorphin, and endomorphin release, whereas 100 Hz EA increase the release of dynorphin. A combination of 2 Hz and 100 Hz EA may induce simultaneous release of all four opioid peptides produce maximal therapeutic effect [29, 30]. Therefore, how to increase therapeutic effect of EA, further study is need. Basic characteristics of age, prostate size, voiding volume, total flow time, Qave, and Qmax were not significantly different between two groups. 

 Uroflowmetry may be used for preoperative routine assessment of BPH patients [12]. The Qmax may predict the degree of obstruction in patients with BPH because there is a negative correlation between Qmax and prostate volume [31]. The Qmax is a reliable and objective measurement to evaluate the symptoms of BPH patients, because there is negative correlation between Qmax and the International Continence Society-BPH (ICS-BPH) symptom score [32]. The ICS-BPH symptom score is obtained by using an ICSmale questionnaire, and has a high level of psychometric validity and reliability [33]. Our results also indicated that IPSS and its subscore and PSA were not significantly different between the EG and the CG. The IPSS is a sensible and reliable method to assess LUTS including emptying symptoms: incomplete emptying, intermittency, weak stream, and straining and storage symptoms: frequency, urgency, and nocturia in BPH patients [8], whereas subscores of IPSS consist of the emptying symptoms score and the storage symptoms score [34]. 

 One report finds that with the IPSS, it is difficult to predict the severity of the symptoms in China [35]. Other reports demonstrate that the correlation is poor between total IPSS and bladder outlet obstruction [36, 37]. Because of bladder outlet obstruction is related to the dysfunction of detrusor [38]. The prostate size is a very weak determinant factor of the severity of symptoms and bladder outlet obstruction, because the bladder outlet obstruction of BPH included both static and dynamic factors that have a relationship to the tension of the prostate smooth muscle [39]. The LUTS are induced by BPH due to obstruction causing detrusor dysfunction, resulting in neural alteration of the bladder and prostate [40]. PSA is important tumor marker of prostate cancer that is used for early detection in patients with BPH and in the management of patients with prostate cancer [15]. EA may partly improve voiding function of patients with BPH. Acupuncture is about twice as effective as sham acupuncture in the treatment of patients with prostatitis/chronic pelvis pain [41]. Acupuncture may work via a somatoautonomic reflex to reduce or increase gastroduodenal motility. In rats, acupuncture in the abdomen, which excites sympathetic nerves through a spinal reflex, reduces gastric motility. Acupuncture in the hind limbs, which excites the vagus nerve via a supraspinal reflex, increases gastric motility [42]. Acupuncture or electrical stimulation may increase the production of endorphins and analgesic effects to generate a neuromodulation to decrease the high tone of the pelvic floor which is induced by bladder dysfunction [43]. The reflexotherapy of EA can improve sensory urgency in BPH patients with transurethral resection [44]. To sum up, EA increase the voiding volume, Qave, and Qmax of BPH patients with LUTS, possibly through many pathways. The neuromodulation of the bladder induced by acupuncture appears to play a critical role.

Although the results of the present study indicated that 2 Hz EA of Zhongji, Guanyuan, ipsilateral Zusanli, and Sanyinjiao might increase the voiding volume, Qave, and Qmax of BPH patients with LUTS, the study still is unclear that these acupoints are essential or stimulation of others acupoints also effective or not. Therefore, further the examination of some other acupoints effect is needed. Acupuncture apply to CV1 (Huiyin), Guanyuan, Sanyinjiao and SP9 (Yinlingquan) 2 weekly sessions for 10 weeks may improve chronic prostatitis/chronic pelvis pain symptoms of adult men [41]. Electrostimulation of Huiyin, CV2 (Qugu), CV4, CV5 (Shimen), BL21 (Weishu), BL23 (Shenshu), BL32 (Ciliao), and auricular points of prostate and external genitalia also can improve LUTS in patients with transurethral resection of the prostate [44]. 

 The present study had some limitations: (1) the small sample size in our trial could result in a lack of statistically significant differences between two groups in the IPSS and its subscore and (2) acupuncture needles were inserted in the subcutaneous region to a depth of 2 mm in the control group, and therefore, we could not exclude the production of different subliminal effect [45, 46]. The adoption of a larger sample size and different study design might have eliminated the two above-mentioned limitations. Although three patients developed ecchymosis in the present study, this ecchymosed disappeared completely, and these patients did not withdraw form the study. We consider EA to be safe.

In conclusion, the clinical improvement of 2 Hz EA was quite limited to the LUTS of patients with BPH; how to increase the therapeutic effect of EA for the LUTS treatment of patient with BPH needs further study.

## Figures and Tables

**Table 1:** Standards for reporting interventions in controlled trials of acupuncture (STRICTA).

Acupuncture rationale	(1) According to the selection of local points, and meridian theory of traditional Chinese medicine
	(2) Classic acupoints: CV3 (Zhongji), CV4 (Guanyuan), ST36 (Zusanli), SP6 (Sanyinjiao)
Needling detail	(1) Single or bilateral acupoints
	(2) Six needles inserted
	(3) Depth of insertion: muscle layer in electroacupuncture (EA) group, and subcutaneous tissue in sham EA group
	(4) Responses elicited: obtain qi and visible twitching of muscle in EA group, and no responses elicited in sham EA group
	(5) Needle stimulation: manual to obtain qi following by electrical stimulation in EA group, and no manual or any electrical stimulation in sham EA group
	(6) Needle retention time: 20 min
	(7) Needle type: stainless steel needles, 2 cun in length, gauge#30, Yu Huang, Taiwan

Treatment regimen	(1) Twice per week for 6 weeks

Cointerventions	(1) None: no herbs, moxibustion, cupping, massage, exercise, advice dietary or lifestyle modification

Practitioner background	(1) License-certificated Chinese medical doctor with more than four years of acupuncture experience

Control intervention	(1) Nil

**Table 2:** Basic character.

	EG (*n* = 18)	CG (*n* = 19)	*P* value
Age (years)	63.2 ± 10.0	59.8 ± 9.0	.399
Prostate volume (mL)	36.0 ± 11.8	38.4 ± 17	.910
IPSS	22.8 ± 4.4	21.4 ± 3.1	.276
Voiding volume (mL)	190.7 ± 109.4	254.6 ± 122.6	.051
Total flow time (sec)	51.8 ± 22.1	55.5 ± 24.6	.706
Average flow rate (mL/sec)	4.0 ± 1.9	4.9 ± 1.8	.131
Maximal flow rate (mL/sec)	9.7 ± 3.2	11.2 ± 2.5	.128
PSA (ng/mL)	2.5 ± 2.2	3.4 ± 3.5	.589

Data present as mean ± standard deviation; EG: electroacupuncture group; CG: sham electroacupuncture group; IPSS: International Prostate Symptom score; PSA: prostate-specific antigen.

**Table 3:** Effect of electroacupuncture on voiding symptoms in patients with benign prostate hyperplasia.

	EG (*n* = 18)	CG (*n* = 19)	*P* value
Voiding volume (mL)			
Baseline	190.7 ± 109.4	254.6 ± 122.6	.051
6 weeks	230.1 ± 107.9	223.4 ± 127.8	.749
Baseline-6 weeks	32.2 ± 104.4	−37.9 ± 120.4	.038*
Total flow time (sec)			
Baseline	51.8 ± 22.1	55.5 ± 24.6	.706
6 weeks	42.5 ± 22.1	52.7 ± 31.5	.380
Baseline-6 weeks	−10.5 ± 30.6	−3.69 ± 26.4	.607
Average flow rate (mL/sec)			
Baseline	4.0 ± 1.9	4.9 ± 1.8	.131
6 weeks	5.3 ± 2.2	4.7 ± 2.3	.309
Baseline-6 weeks	1.2 ± 1.6	−0.22 ± 2.7	.026*
Maximal flow rate (mL/sec)			
Before	9.7 ± 3.2	11.2 ± 2.5	.128
6 weeks	11.8 ± 4.7	10.9 ± 4.2	.792
Baseline-6weeks	2.3 ± 3.7	−0.3 ± 4.3	.030*

Data present as mean ± standard deviation; EG: electrooacupuncture group; CG: sham electroacupuncture group; Baseline: before electracupuncture; 6 weeks: at finishing 6 consecutive weeks total twelve times electroacupuncture; Baseline-6 weeks: the difference between baseline and at 6 weeks; *P < .05 EG versus CG.

**Table 4:** Effect of electroacupuncture on lower urinary symptoms and prostate specific antigen in patients with benign prostate hyperplasia.

	EG (*n* = 18)	CG (*n* = 19)	*P* value
IPSS			
Baseline	22.8 ± 4.4	21.4 ± 3.1	.276
3 weeks	19.5 ± 4.3	18.7 ± 4.0	.640
6 weeks	18.0 ± 3.8	18.2 ± 4.5	.932
Baseline-3weeks	−3.2 ± 2.4	−2.6 ± 3.2	.314
Baseline-6weeks	−4.4 ± 2.6	−3.05 ± 3.8	.175
PSA (ng/mL)			
Baseline	2.5 ± 2.2	3.4 ± 3.5	.589
6 weeks	2.4 ± 2.1	3.4 ± 3.7	.573
Baseline-6 weeks	−0.12 ± 0.54	−0.03 ± 1.08	.573

Data present as mean ± standard deviation; EG: electroacupuncture group; CG: sham electroacupuncture group; IPSS: international prostate symptom score; PSA: prostate-specific antigen; Baseline: before electroacupuncture; 3 weeks: at finishing 3 consecutive weeks total six times electroacupuncture; 6 weeks: at finishing 6 consecutive weeks total twelve times electroacupuncture; Baseline-3 weeks: the difference between baseline and at 3 weeks; Baseline-6 weeks: the difference between baseline and at 6 weeks.

**Table 5:** Effect of electroacupuncture on subscore of IPSS in patients with benign prostate hyperplasia.

	EA (*n* = 18)	CG (*n* = 19)	*P* value
Incomplete emptying			
Baseline	3.4 ± 1.2	3.4 ± 1.2	.766
3 weeks	2.9 ± 1.3	3.0 ± 0.9	.799
6 week	2.8 ± 1.3	3.1 ± 0.9	.443
ΔBaseline-3 weeks	0.56 ± 1.04	0.37 ± 0.60	.94
▲Baseline-6 weeks	0.67 ± 1.03	0.32 ± 0.67	.461
Frequency			
Baseline	3.3 ± 1.1	3.4 ± 1.1	.834
3 weeks	2.9 ± 0.9	2.9 ± 1.2	.940
6 weeks	2.7 ± 1.3	2.7 ± 1.2	.940
ΔBaseline-3 weeks	0.44 ± 0.62	0.47 ± 0.77	.730
▲Baseline-6 weeks	0.67 ± 0.59	0.62 ± 1.01	.298
Intermittency			
Baseline	3.1 ± 1.0	3.0 ± 1.2	.675
3 weeks	2.7 ± 1.0	2.9 ± 1.2	.499
6 weeks	2.7 ± 1.0	2.8 ± 1.2	.851
ΔBaseline-3 weeks	0.39 ± 0.50	0.05 ± 0.62	.159
▲Baseline-6 weeks	0.35 ± 0.49	0.21 ± 0.63	.594
Urgency			
Baseline	2.8 ± 1.5	2.7 ± 1.1	.938
3 weeks	2.5 ± 1.2	2.3 ± 1.1	.707
6 weeks	2.3 ± 1.1	2.4 ± 1.2	.753
ΔBaseline-3 weeks	0.33 ± 0.97	0.47 ± 0.90	.707
▲Baseline-6 weeks	0.56 ± 1.04	0.32 ± 1.0	.685
Weak stream			
Baseline	3.4 ± 1.0	3.2 ± 1.0	.490
3 weeks	3.1 ± 1.0	2.8 ± 0.8	.518
6 weeks	3.1 ± 0.9	2.7 ± 0.7	.245
ΔBaseline-3 weeks	0.28 ± 0.46	0.32 ± 0.58	1.000
▲Baseline-6 weeks	0.33 ± 0.59	0.47 ± 0.70	.775
Straining			
baseline	3.3 ± 1.0	3.2 ± 0.7	.705
3 weeks	2.8 ± 1.0	2.9 ± 0.7	.753
6 weeks	2.7 ± 0.9	2.8 ± 0.7	.707
ΔBaseline-3 weeks	0.44 ± 0.62	0.26 ± 0.65	.443
▲Baseline-6 weeks	0.56 ± 0.70	0.37 ± 0.83	.518
Nocturia			
Baseline	2.6 ± 1.0	2.6 ± 1.2	.961
3 weeks	2.1 ± 0.9	2.0 ± 1.2	.893
6 weeks	1.6 ± 0.7	2.0 ± 1.0	.358
ΔBaseline-3 weeks	0.50 ± 0.62	0.32 ± 0.7	.988
▲Baseline-6 weeks	1.0 ± 0.84	0.58 ± 0.9	.271

Data present as mean ± standard deviation; EG: electroacupuncture group; CG: sham electroacupuncture group; Baseline: before electroacupuncture; 3 weeks: at finishing 3 consecutive weeks total six times electroacupuncture; 6 weeks: at finishing 6 consecutive weeks total twelve times electroacupuncture; Δ: difference between baseline and at 3 weeks; ▲: difference between baseline and at 6 weeks.
